# Proteolytic Regulation of the Lectin-Like Oxidized Lipoprotein Receptor LOX-1

**DOI:** 10.3389/fcvm.2020.594441

**Published:** 2021-01-20

**Authors:** Torben Mentrup, Florencia Cabrera-Cabrera, Bernd Schröder

**Affiliations:** Institute for Physiological Chemistry, Technische Universität Dresden, Dresden, Germany

**Keywords:** LOX-1, atherosclerosis, protease, intramembrane protease, ectodomain shedding, signal transduction, endothelial dysfunction

## Abstract

The lectin-like oxidized-LDL (oxLDL) receptor LOX-1, which is broadly expressed in vascular cells, represents a key mediator of endothelial activation and dysfunction in atherosclerotic plaque development. Being a member of the C-type lectin receptor family, LOX-1 can bind different ligands, with oxLDL being the best characterized. LOX-1 mediates oxLDL uptake into vascular cells and by this means can promote foam cell formation. In addition, LOX-1 triggers multiple signaling pathways, which ultimately induce a pro-atherogenic and pro-fibrotic transcriptional program. However, the molecular mechanisms underlying this signal transduction remain incompletely understood. In this regard, proteolysis has recently emerged as a regulatory mechanism of LOX-1 function. Different proteolytic cleavages within the LOX-1 protein can initiate its turnover and control the cellular levels of this receptor. Thereby, cleavage products with individual biological functions and/or medical significance are produced. Ectodomain shedding leads to the release of a soluble form of the receptor (sLOX1) which has been suggested to have diagnostic potential as a biomarker. Removal of the ectodomain leaves behind a membrane-bound N-terminal fragment (NTF), which despite being devoid of the ligand-binding domain is actively involved in signal transduction. Degradation of this LOX-1 NTF, which represents an athero-protective mechanism, critically depends on the aspartyl intramembrane proteases Signal peptide peptidase-like 2a and b (SPPL2a/b). Here, we present an overview of the biology of LOX-1 focusing on how proteolytic cleavages directly modulate the function of this receptor and, what kind of pathophysiological implications this has in cardiovascular disease.

## Introduction

Lipid deposition and foam cell formation are hallmarks in the developement of atherosclerotic plaques (1). Chemically modified lipoproteins, e.g., by oxidation, which are no longer recognized by the LDL receptor but instead bind to different scavenger receptors play a particular role in this context (2). Oxidized LDL (oxLDL) has pro-atherogenic properties and increased plasma levels have been reported to be associated with coronary heart disease (3). oxLDL exerts effects on all cell types in the vascular wall and is especially taken up by macrophages, where it is associated with foam cell formation (4). Several oxLDL receptors have been reported including the class A scavenger receptors and CD36 (2). The Lectin-like oxLDL receptor 1 (LOX-1) was identified by an expression cloning approach aiming to reveal endothelial receptors for oxLDL (5). Since then, further research has highlighted this protein as a key player in cardiovascular pathophysiology and disease, which is critically linked to its ability to bind and mediate internalization of oxLDL.

**Figure 1:**
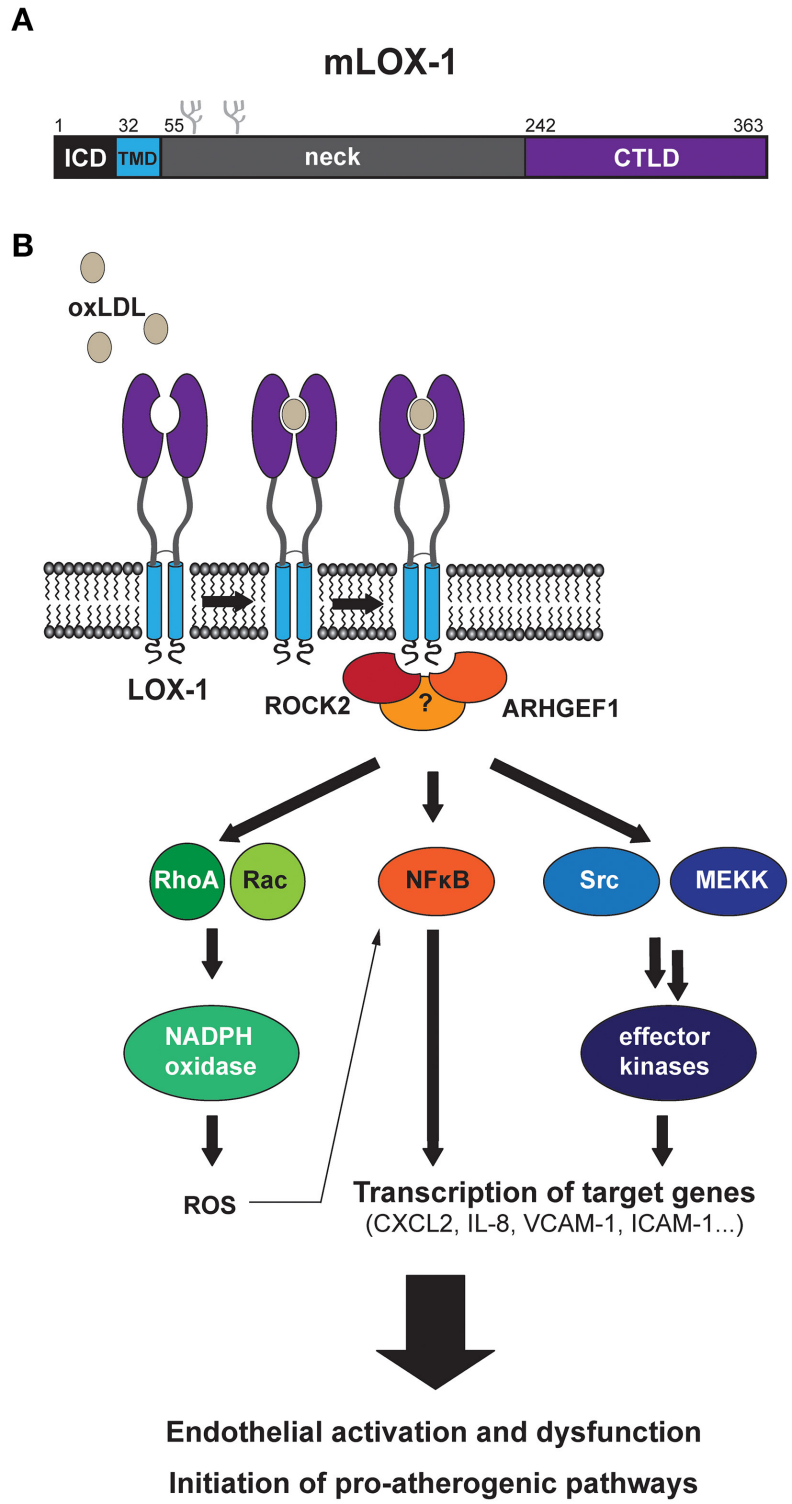
Current model of LOX-1-dependent signal transduction. **(A)** Schematic overview of the protein domains of murine LOX-1 indicating positions of the intracellular domain (ICD), the transmembrane domain (TMD), the neck domain and the ligand binding C-type lectin domain (CTLD). The overall domain structure of human LOX-1 is similar to that of murine LOX-1. **(B)** Binding of oxLDL to LOX-1 at the cell surface induces various signaling responses. These activate endothelial cells, can lead to endothelial dysfunction and finally promote the development of atherosclerotic plaques. The initial mechanisms by which LOX-1 initiates signaling are only partly understood. An interaction of LOX-1 with the cytosolic proteins ROCK2 and ARHGEF1 was suggested to be involved in triggering signal transduction. However, it is not yet clear how precisely these proteins couple to downstream pathways and in light of the pleiotropic response it seems likely that further mechanisms are involved here. Different downstream axes have been described, which may be significantly interconnected. These include the GTPases RhoA and Rac, which have been linked with oxLDL-induced production of reactive oxygen species (ROS). Furthermore, activation of the NFκB pathway as well as other effector kinases including ERK1/2 and p38 MAP kinases has been reported. Several transcriptional targets, which are upregulated by oxLDL-induced LOX-1 activation, were identified such as different cytokines/chemokines, adhesion molecules, and members of the matrix metalloprotease family.

LOX-1 belongs to the family of C-type lectin receptors (6). Members of this family share a conserved carbohydrate-binding C-type lectin domain (CTLD) and several of them act as pattern recognition receptors in the innate immune system. LOX-1 is a single-span, type II-oriented transmembrane protein with a rather short intracellular domain (ICD) of about 30 residues (Figure 1A). The transmembrane segment and the extracellular CTLD are connected by a neck domain (7). Human LOX-1 forms homo-dimers that are linked by a disulfide bridge in the neck domain. Furthermore, LOX-1 seems to be able to form even larger non-covalent oligomeric complexes (8).

Based on the determined crystal structure (7), the LOX-1 CTLD contains all of the main secondary structure elements characteristic for members of the C-type lectin-like superfamily. Beyond oxLDL, LOX-1 can interact with a variety of other ligands (9). These include different forms of chemically modified lipoproteins, but also ligands with no obvious structural similarity to lipoproteins like the C-reactive protein (10) or extracellularly released heat shock proteins like Hsp70 (11). Furthermore, several cellular ligands of LOX-1 have been reported, among these are activated platelets (12), leukocytes (13), aged/apoptotic cells (14), and also bacteria (15).

Though initially cloned from a cDNA-derived library from endothelial cells (5), the expression of LOX-1 is not limited to this cell type. LOX-1 is also expressed in vascular smooth muscle cells (16, 17), differentiated macrophages (18), dendritic cells (DCs) (19), B cells (19) neurons (20), and platelets (21). Based on its presence in the vasculature, the function of LOX-1 has been studied in the context of cardiovascular pathophysiology. Nevertheless, LOX-1 has also been found to be important in other processes such as immunity (11). It may be anticipated that further functions of LOX-1 remain to be discovered.

## Cellular and Pathophysiological Functions of LOX-1

### Signal Transduction of LOX-1

LOX-1 was identified based on its ability to bind ox-LDL raising the question what the cellular consequences of ligand binding are. It was observed that LOX-1 can mediate internalization of the bound cargo (5, 14, 22). However, interaction of LOX-1 with its respective ligands induces additional biological effects, which have been mainly analyzed for oxLDL (Figure 1B). It was found that binding of oxLDL to LOX-1 in endothelial cells activates the NFκB pathway (23) as well as the production of reactive oxygen species (ROS) (24). In addition, also the activation of ERK1/2 and/or p38 MAP kinases downstream of LOX-1 has been reported in different cell types (25–28). In contrast, LOX-1 downregulates phosphorylation and activity of the kinase Akt, which has been linked to a decreased activity of the endothelial nitric oxide (NO) synthase (28). LOX-1 can also stimulate the small GTPases RhoA and Rac1, which was dependent on the matrix metalloproteinase MT1-MMP (29).

Based on this complex LOX-1 signaling response, the question arises how these diverse pathways can be activated at the molecular level and which may be critical determinants of the LOX-1 protein coupling the receptor to these downstream pathways. Altogether, the receptor-proximal events of LOX-1 signaling remain poorly defined to date. The LOX-1 ICD does not harbor any documented signaling motif. Dimerization of LOX-1 was found to be critical for MAP kinase activation since heterodimerization with a dominant-negative receptor carrying mutations in the extracellular domain significantly reduced the ox-LDL induced signaling response (30). Interestingly, binding of oxLDL to the receptor complexes did not seem to be compromized by this mutation. These findings indicate that dimerization may be important for the recruitment of potential cytosolic adaptor proteins. As two potential interaction partners, Mattaliano et al. identified ARHGEF1 and ROCK2, which are involved in the Rho signaling pathway (31). Whereas ARHGEF1 bound to LOX-1 in a constitutive manner, ROCK2 was only recovered in receptor complexes after oxLDL stimulation of the cells. Inhibition of ROCK2 reduced oxLDL- induced activation of the NFκB pathway and also impaired downstream effects as e.g., the production of certain cytokines (31). These findings identified ROCK2 as an important mediator of LOX-1 signaling.

### Downstream Effectors of LOX-1 Activation

In contrast to the poorly understood signaling mechanism, LOX-1 downstream targets have been characterized more extensively. An important function of the endothelium is the production of NO, which promotes vasodilation and also counteracts inadvertent thrombocyte activation. LOX-1 can lead to a reduced production of NO (29, 32). In addition to ROS production, LOX-1 activation significantly modulates the expression of several target genes. Microarray-analysis of oxLDL-treated HAECT cells overexpressing LOX-1 identified significant transcriptional changes associated with inflammation, cell adhesion and different signal transduction pathways (33), including upregulation of several cytokines like interleukin-8, CXCL2, CXCL3 and colony-stimulating-factor 3. LOX-1 mediated downstream effects can similarly be observed in fibroblasts by overexpressing the receptor. This leads to enhanced expression of the leukocyte adhesion molecules ICAM-1 and VCAM-1 as well as of MMP1 (34).

### Contribution of LOX-1 to Cardiovascular Disease

With regard to endothelial cells, consequences of LOX-1 signaling like the reduced NO production and enhanced production of cytokines and adhesion molecules contribute significantly to the development of endothelial dysfunction. This state is considered to be one of the initiating events for atherosclerotic plaque formation (35). Along this line, transgenic overexpression of bovine LOX-1 in Apolipoprotein E (ApoE) knock out mice, which received a high fat diet (HFD), was associated with an accumulation of oxLDL in coronary arteries (36). In addition, expression of adhesion molecules and recruitment of macrophages were increased leading to larger atheroma-like lesions. Similarly, ApoE knock out mice subjected to adenoviral overexpression of LOX-1 primarily targeting the ECs of the carotid arteries displayed increased plaque coverage (37). Furthermore, when transgenic mice overexpressing LOX-1 specifically in ECs received a HFD, accelerated formation of fatty streaks, an increased expression of adhesion molecules and enhanced macrophage recruitment were observed (38).

In agreement with overexpression models, constitutive deletion of LOX-1 resulted in reduced atherogenesis (39). Ablation of LOX-1 limited the production of inflammatory and oxidative stress related signals, thus preserving endothelial cell function. Furthermore, plaque areas in aortas of LOX-1 null mice were significantly smaller and thickness of the intima was also significantly reduced (39). Deletion of LOX-1 also led to reduced collagen deposition in vessel walls of LDLR-knock out mice (40).

Beyond atherosclerosis, LOX-1 can also have an impact on the course of ischemic complications. In hearts of rats subjected to cardiac ischemia-reperfusion, a pronounced upregulation of LOX-1 was observed. Administration of a LOX-1 antibody reduced overall tissue damage (41). In agreement, LOX-1 KO mice showed improved left ventricle function in comparison to wild type mice in a similar set-up (42). Oxidative stress and collagen accumulation were also significantly decreased in these mice (42).

In a rat model of cerebral ischemia the expression of LOX-1 was dramatically increased in the core lesion site (43). LOX-1 deficient mice subjected to focal cerebral ischemia showed a reduced degree of brain injury, which was recapitulated with application of a LOX-1 antibody prior to stroke induction (44). Mice overexpressing LOX-1 in endothelial cells showed increased cerebral damage and neurologic deficits after a transient middle cerebral artery occlusion, while application of LOX-1 siRNA had a protective effect (45).

Chronic arterial hypertension can result in cardiac remodeling, which is enhanced by Angiotensin II (Ang II) (46). Ang II promotes the expression of LOX-1 via AT1R (47), which in turn stimulates expression of AT1R (48) and also associates with and activates the AT1R (49). Upon chronic administration of Ang II, cardiac hypertrophy and remodeling were reduced in LOX-1 knockout mice (50). Deletion of LOX-1 also resulted in reduced ROS generation and MAPK activation. While in WT mice Ang II treatment led to an increased expression of AT1R, this was much less pronounced upon LOX-1 deficiency, supporting a positive feedback loop between Ang II, AT1R, ROS, and LOX-1 (50).

## Regulation of LOX-1 Function

**Figure 2:**
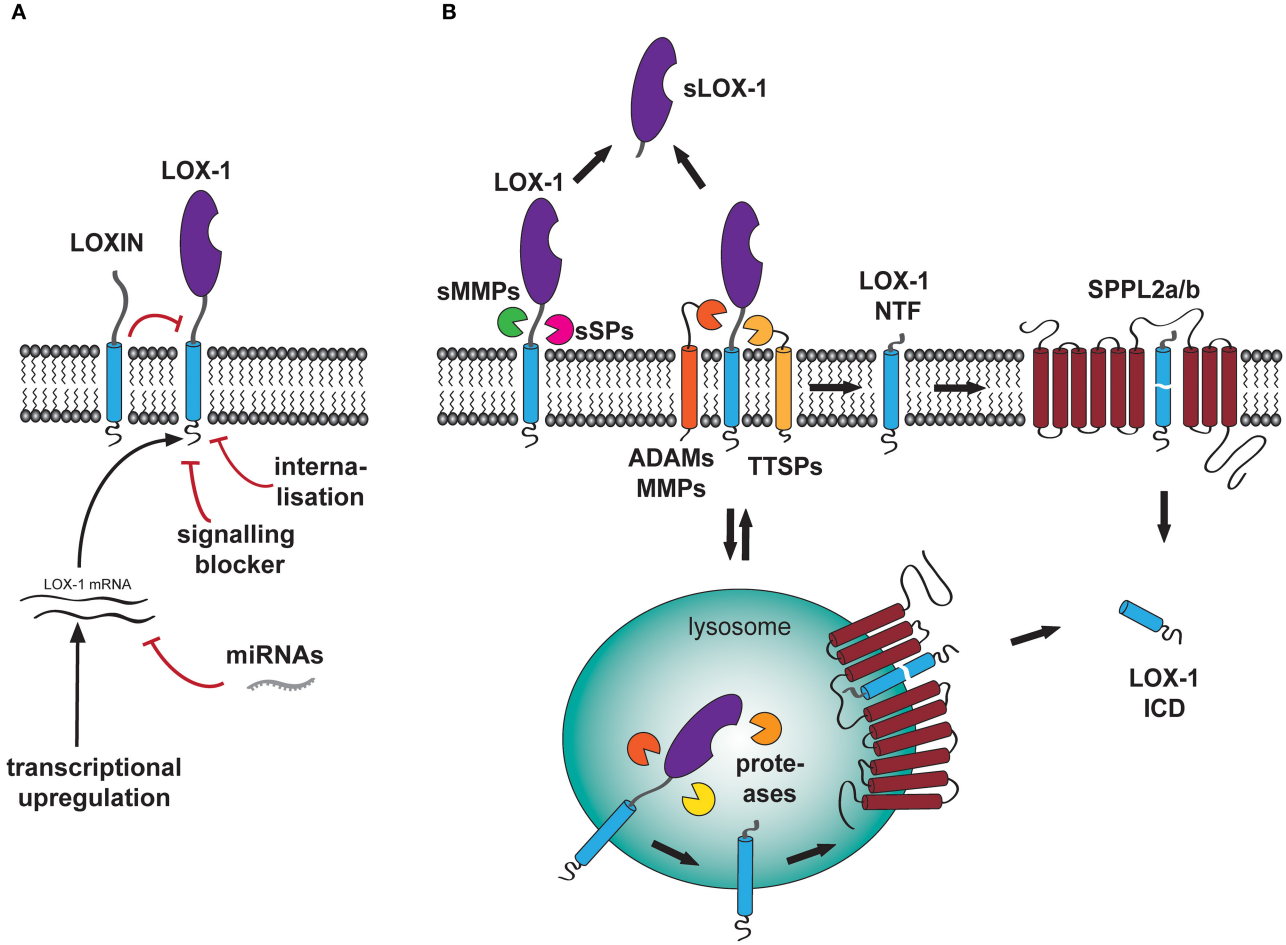
Regulation of LOX-1 surface levels and function. **(A)** At a non-proteolytic level, LOX-1 functions can be regulated by several processes including internalization, activity of signaling blocking molecules (SIRT1) as well as transcriptional upregulation of the receptor expression. The latter aspect can be negatively affected by binding of miRNAs to the LOX-1 mRNA. **(B)** At the cell surface, full length LOX-1 can be processed by a plethora of proteases including the membrane-bound metalloproteases ADAM10 and ADAM17 as well as soluble matrix metalloproteinases MMP-1 and MMP-2. Furthermore, uncharacterized serine proteases, which might belong to either the family of type II transmembrane serine proteases (TTSP) or act as soluble serine proteases (sSP), might process LOX-1 in its ectodomain releasing a soluble fragment of the receptor (sLOX-1) to the blood stream. In addition, internalized LOX-1 is also degraded in the lysosome by pH-sensitive proteases. Both processes produce membrane-bound LOX-1 N-terminal fragments (NTF), that are subsequently processed by the intramembrane-cleaving proteases SPPL2a and SPPL2b. This cleavage results in the cytosolic liberation of the LOX-1 intracellular domain (ICD).

Due to its involvement in the development and progression of cardiovascular disease, regulation of LOX-1 has been under intensive investigation. One central aspect of regulating the receptor activity is the control of the availability of signaling-competent receptor molecules at the cell surface. In principle, this can be achieved by several independent mechanisms including transcriptional regulation of receptor synthesis, modulation of activity of already existing surface receptors by interaction with regulatory co-factors, modulation of signaling cascades or elimination of surface receptors by either internalization (Figure 2A) or proteolytic turnover (Figure 2B).

### Regulation of LOX-1 Transcription

While so far only little is known about intracellular processes regulating LOX-1-dependent signaling, our current understanding of LOX-1 transcription is much more advanced. In agreement with its low expression in healthy vasculature, expression of LOX-1 and therefore also surface levels of the oxLDL receptor are significantly upregulated by various pro-inflammatory or pro-atherogenic stimuli as reviewed in Hermonat et al. (51). Among others, these stimuli include acute phase-inducing cytokines like IL-1α/β, IL-6, and TNFα, but also disease-associated factors like bacterial lipopolysaccharides and elevated glucose levels (52–55). Importantly, also the primary LOX-1 ligand, oxLDL, leads to an upregulation of its own receptor based on the induced signaling (56). This can induce a self-perpetuating positive feedback loop and a vicious cycle since increased LOX-1 levels support pathophysiological cascades finally leading to cardiovascular diseases.

On a molecular view, the upregulation of LOX-1 could be attributed to the presence of several transcription factor binding sites in the *Olr1* promoter. In the rat *Olr1* promoter, binding sites for the GATA transcription factor family, Oct-1 and AP-1 were identified (53). As shown by EMSA experiments employing an 106 bp fragment of the rat *Olr1* promoter, Oct-1 specifically binds to this region in the presence of oxLDL (57). In addition, NFκB could be demonstrated to activate transcription of the Olr1 gene (55, 58). Interestingly, Oct-1 and NFκB also represent downstream targets of LOX-1 signaling axes and therefore might be responsible for the strong induction of LOX-1 expression in the presence of oxLDL (24, 59). Furthermore, LOX-1 transcription is induced upon elevated shear stress as well as endothelial cell stimulation with angiotensin II. This is facilitated by the presence of a shear stress element in the rat *Olr1* promoter (53).

### Regulation of LOX-1 by miRNAs

Cellular LOX-1 levels are also controlled post-transcriptionally. Especially in non-activated vascular cells, LOX-1 mRNA levels are actively suppressed by microRNAs (miRNAs). miRNAs are short, single stranded non-coding RNA molecules that regulate gene expression by binding to mRNAs that are subsequently targeted for degradation (60). This interaction is sequence-specific and therefore allows targeted suppression of gene expression. In the case of LOX-1, three different miRNAs have been identified (miRNA-98, mir-let-7g, miR-590-5p), that downregulate LOX-1 mRNA by directly binding to the 3' UTR regions of the *Olr1* transcript. As shown in aortic smooth muscle cells and HUVEC but also by use of ApoE-deficient mouse models of atherosclerosis, these miRNAs downregulate LOX-1 expression (61–65). In turn, increased LOX-1 expression also represses transcription of these miRNAs thereby leading to an enhanced auto-activation.

### Sirtuin-1 as Negative Effector of LOX-1

Another negative regulator of LOX-1 is Sirtuin-1 (SIRT1), which acts as a major protein deacetylase (66). As demonstrated by Stein et al. (67), SIRT1 is able to repress LOX-1-dependent formation of foam cells. In ApoE-deficient mice, depletion of one SIRT1 allele caused a significant increase in plaque burden and oxLDL uptake into macrophages. Indeed, the function of SIRT1 in macrophages appeared to be relevant for the elevated atherogenic response since restoration of Sirt1^+/−^ mice with wild type bone marrow rescued the observed phenotype. Reduction of SIRT1-dependent deacetylation of NFκB components (RelA/p65) was identified as the underlying molecular mechanism, which also caused a downregulation of LOX-1 surface levels on macrophages (67). In agreement with this, inhibition of SIRT1 resulted in increased oxidative stress as well as an elevated inflammatory response in patients with coronary artery disease highlighting the *in vivo* relevance of the negative regulation of LOX-1 activity by SIRT1 (67).

### LOX-1 Regulation by Internalization

LOX-1 surface levels are also controlled by internalization of the receptor. Interestingly, this process is considered to occur constitutively and independent of stimulation with oxLDL as a *bona fide* LOX-1 ligand (68, 69). In HeLa cells, co-transfection of tagged LOX-1 with a dominant-negative mutant of Dynamin-2 (K44A) completely blocked receptor endocytosis. However, reducing the expression of the clathrin heavy chain (CHC17) or the transport adaptor protein AP-2 by siRNA had no effect, arguing for a dynamin-dependent but clathrin-independent mechanism (68). In the same study, a conserved DDL/P motif was identified in the N-terminus of LOX-1 which is required for its efficient endocytosis. Mutation of D4 and L6 of human LOX-1 to alanine caused a reduction of internalization efficiency of about 40% while introduction of the D5A mutant almost completely abolished constitutive receptor internalization (68).

### LOXIN Negatively Regulates LOX-1 Function

Plasma membrane localization and ligand binding capacity of LOX-1 are also regulated by another mechanism. LOXIN is a splice isoform of LOX-1 that lacks exon 5 (70). This leads to a stop codon in the open reading frame and a shortened protein devoid of two thirds of the CTLD and therefore deficient in oxLDL-binding (71). LOXIN expressed on its own does not efficiently reach the cell surface. Importantly, LOXIN can form hetero-oligomers with full length LOX-1, which seem to be non-functional (71). Furthermore, LOXIN is able to diminish LOX-1 surface levels (71). In agreement, LOXIN overexpression in human endothelial progenitor cells reduced oxLDL uptake and protected the cells from oxLDL-induced apoptosis (72). Also *in vivo* co-expression of LOXIN significantly attenuated the atherogenic effect of LOX-1 overexpression in carotid arteries of mice (37). Though it is not clear yet if LOXIN levels are subjected to any physiological regulation, these findings further confirm that LOX-1 complex formation is an important prerequisite for its function and a potential mode of regulation. This is also supported by genetic findings as the LOXIN/full length LOX-1 ratio was affected by certain intronic SNPs, which are associated with myocardial infarction (70). High LOXIN expression was observed in monocyte-derived macrophages from subjects carrying the “non-risk” haplotype, suggesting that the LOXIN isoform has a protective role in cardiovascular diseases (70).

### Regulation of LOX-1 by Lysosomal Proteases

LOX-1 surface levels and signaling capacity are also intensively controlled by proteolysis (Figure 2B). On the one hand, LOX-1 is degraded in lysosomal compartments following its internalization. Upon overexpression of tagged murine LOX-1, the receptor was not only localized at the plasma membrane of HeLa cells but also showed co-localization with the lysosomal marker LAMP2 (69). Processing of LOX-1 in these compartments was sensitive to the vacuolar ATPase inhibitor bafilomycin a1 indicating that pH-sensitive proteases, which have not been further characterized, facilitate the degradation of full length LOX-1 in this context (69). Internalization-dependent degradation has been demonstrated for other members of the C-type lectin receptor family (73, 74) highlighting intracellular degradation as a major regulator of CTLR receptor function.

### Regulation of LOX-1 by Ectodomain Shedding

In addition to lysosomal degradation, many single-pass transmembrane receptors undergo proteolytic ectodomain release, which is also described as ectodomain shedding. In this process, a soluble or transmembrane protease facilitates the controlled cleavage of a membrane-bound substrate molecule. In the case of transmembrane receptors, proteolytic processing typically leads to the release of the extracellular ligand binding domain thereby intensively regulating the signaling capacities of the receptor expressing cell (75). The regulatory potential of this pathway for receptor activity has been demonstrated for many examples including Notch1, the EGFR and other receptor tyrosine kinases (75).

Along this line, Murase et al. identified a soluble N-terminally truncated version of bovine LOX-1 in the supernatants of TNFα-stimulated bovine aortic endothelial cells as well as stably transfected CHO-K1 cells (76). As demonstrated with biotinylation experiments, surface expression of LOX-1 preceded the release of soluble LOX-1 (sLOX-1) strongly arguing for a proteolytic liberation of this protein fragment from the membrane-bound full length receptor (76). Two cleavage sites were identified in bovine LOX-1 between R86 and S87 as well as K89 and S90 (76). A similar cleavage site was later described for human LOX-1 between R88 and Q89 (77) suggesting a conserved mechanism for LOX-1 ectodomain shedding.

While the existence of a soluble LOX-1 fragment was subsequently verified in several other model systems and also human patients (76, 78–81), the protease(s) responsible for the processing of full length LOX-1 is still under intense debate. Employing different experimental setups and cellular models, soluble as well as transmembrane serine and metalloproteases have been suggested to mediate sLOX-1 release. In their initial description of sLOX-1, Murase et al. almost completely blocked sLOX-1 release from CHO-K1 cells stably overexpressing bovine LOX-1 by the administration of the broadband serine protease inhibitor PMSF. However, other serine protease inhibitors like leupeptin and TLCK only had minor effects or in case of aprotinin did not prevent LOX-1 processing at all. Application of cysteine or aspartic protease inhibitors also did not affect sLOX-1 release (76). The hypothesis that indeed a PMSF-sensitive serine protease is involved was further supported by another study demonstrating that CRP-stimulated release of soluble LOX-1 from TNFα-activated THP-1 cells was efficiently prevented by this inhibitor (78).

### Metalloproteinases Contribute to sLOX-1 Liberation

However, despite this clear evidence, there is also a substantial amount of data suggesting metalloproteases either from the A disintegrin and metalloproteinase (ADAM) or matrix metalloproteinase (MMP) family act as major sheddases of LOX-1. Zhao et al. suggested the metalloprotease ADAM17 as sheddase of LOX-1 (78). Blocking ADAM17 activity by either application of the broadband metalloprotease inhibitor TAPI-1 or knockdown of ADAM17 expression by siRNA significantly reduced sLOX-1 release from activated THP-1 cells (78). A similar effect was observed in macrophages derived from patients with acute coronary syndrome (ACS) (78). ADAM17-mediated release of sLOX-1 was further dependent on intracellular ROS generation as demonstrated by application of antioxidant N-acetylcysteine and blockage of ROS production by the NADPH oxidase blocker apocyanin (78). This is in line with the notion that ADAM17 expressing cells require activation and phosphatidylserine exposure on the outer leaflet of the plasma membrane for full enzymatic activity of the metalloprotease (82). Intracellular toxicity of ROS generated by NADPH oxidase therefore might contribute to ADAM17 activation and increased LOX-1 shedding. In addition to ADAM17, also ADAM10 was suggested to process LOX-1 in its ectodomain. As demonstrated using HEK cells deficient for ADAM10, ADAM17 or both proteases which overexpressed tagged murine LOX-1, ADAM17 was dispensable for release of sLOX-1 (69). In contrast, absence of ADAM10 completely abolished presence of sLOX-1 in conditioned media of these cells. Furthermore, sLOX-1 release could only be stimulated by the Ca^2+^ ionophore ionomycin, which represents a classical activator of ADAM10, but not with the ADAM17 activating compound phorbol-12-myristate-13-acetate (PMA) (69). Similar findings were obtained in HEK cells overexpressing human LOX-1 in which LOX-1 shedding was induced by administration of interleukin-18 (83). In this cell system, overexpression of ADAM10 cDNA together with LOX-1 upregulated sLOX-1 levels while interfering with ADAM10 expression in these cells by siRNA decreased it (83). Indeed, IL-18 also stimulated sLOX-1 levels in a mouse model overexpressing human LOX-1 by about factor 2 establishing it as a relevant regulator of sLOX-1 shedding *in vivo*. A further hint toward a dominant role of ADAM10 is provided by analysis of the reported LOX-1 cleavage sites in proximity to the transmembrane domain of the receptor. In bovine and human LOX-1, these are located after basic arginine or lysine residues, respectively (76, 77). Arginine is indeed favored by ADAM10, but not ADAM17 at this position (84).

In addition to ADAM proteases, also matrix metalloproteinases (MMPs) were suggested to be implicated in shedding of LOX-1. MMPs are critical regulators of extracellular matrix composition and therefore contribute to remodeling of vasculature and plaque formation under pathological conditions (85). Interestingly, MT1-MMP, which is one of the collagen degrading enzymes in atherosclerotic plaques and significantly contributes to pathology in cardiovascular disease (86, 87), has been demonstrated to interact with LOX-1 in human aortic endothelial cells (29). However, cleavage of LOX-1 by MT1-MMP was not analyzed. Furthermore, LOX-1 induces secretion of MMP1 and the related MMP2 and MMP9 in human endothelial EA.hy926 cells (88). While in this study no direct cleavage of LOX-1 by any of these proteases was demonstrated, LOX-1 shedding was sensitive to the broadband metalloprotease inhibitor GM6001 (88). In addition, incubation of LOX-1 overexpressing Cos cells with recombinant soluble MMP1 and MMP2 significantly decreased binding of fluorescently labeled oxLDL (88) supporting a role for these proteases in the release of the LOX-1 ligand binding domain. However, how these proteases contribute to the release of LOX-1 *in vivo* especially under pathophysiological conditions remains to be evaluated.

### LOX-1 Is Regulated by Intramembrane Proteolysis

While research so far focused on the generation of sLOX-1, recently first insights were obtained regarding the fate of the membrane-bound N-terminal fragment (NTF) that is generated during lysosomal degradation and ectodomain shedding of the receptor (69). The existence of a larger glycosylated and a smaller non-glycosylated LOX-1 NTF which were termed NTF1 or NTF2, respectively, was demonstrated in HEK cells overexpressing murine LOX-1. Under endogenous conditions in immortalized murine aortic endothelial cells but also in total lysates of murine aortic vessels only NTF2 was detected. Interestingly, in LDLR-deficient mice subjected to a high fat diet LOX-1 NTF levels increased by almost 2.5-fold (69). LOX-1 NTFs reside at the plasma membrane and the endolysosomal compartment, where they are further processed by the intramembrane proteases Signal peptide peptidase-like (SPPL) 2a and 2b (69). Like presenilins, which represent the catalytically active part of the γ-secretase complex involved in Notch signaling and Alzheimer's disease (89), these proteases belong to the family of aspartic intramembrane proteases that process their substrate molecules in the hydrophobic environment of the lipid bilayer (90–92). This processing event leads to the cytosolic liberation of a LOX-1 intracellular domain (LOX-1 ICD), which so far only could be detected upon overexpression of the receptor. Importantly, if the intramembrane cleavage is blocked, uncleaved LOX-1 NTFs accumulate in the membrane. While deficiency of either SPPL2a or SPPL2b only mildly increased LOX-1 NTF levels in mice, absence of both proteases in SPPL2a/b double-deficient mice clearly elevated NTF levels, demonstrating that intramembrane proteolysis of LOX-1 also occurs *in vivo* (69).

## Functions of LOX-1 Proteolytic Cleavage Fragments

**Figure 3:**
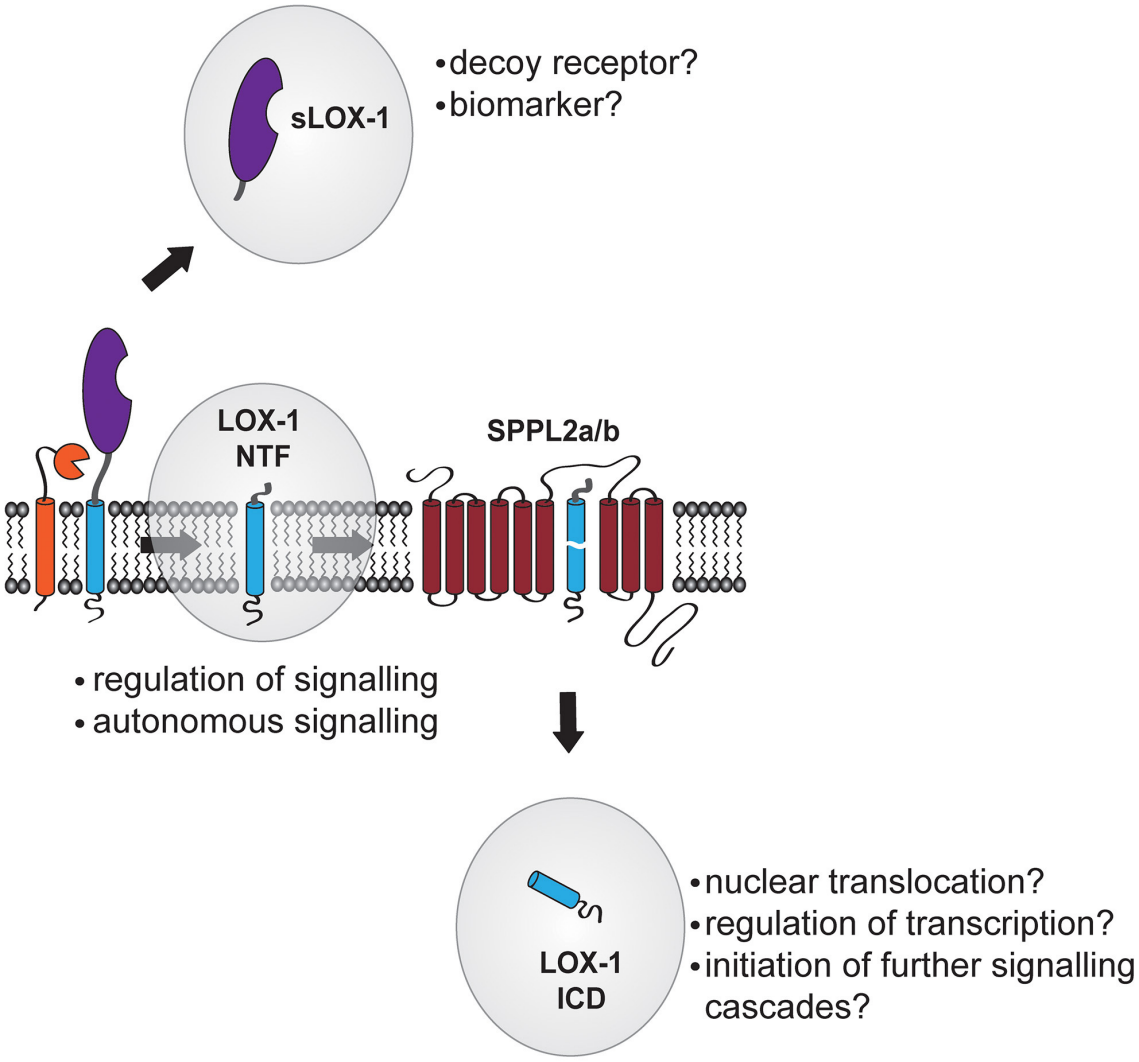
Potential functions of LOX-1 cleavage products. After its proteolytic liberation from the membrane into the blood stream, sLOX-1 might function as decoy receptor. In addition, levels of sLOX-1 in the blood stream of patients might be a novel biomarker for diagnosis of cardiovascular disease. In contrast, the membrane-bound LOX-1 N-terminal fragment (NTF) acts as regulator of both ligand-dependent and -independent signal transduction of LOX-1 increasing the pro-atherogenic signaling of the receptor. Functions of the LOX-1 intracellular domain (ICD) released by SPPL2-mediated intramembrane proteolysis are not known so far. However, this cytosolic fragment might migrate to the nucleus and alter gene transcription of target genes or participate in the regulation of cytosolic signaling cascades.

During proteolytic processing of LOX-1, three major cleavage fragments occur (Figure 3): [1] Ectodomain shedding generates a soluble LOX-1 ectodomain (sLOX-1) that is released to the extracellular compartment and was detected in human patients as well as laboratory animals. [2] By shedding and lysosomal degradation of LOX-1 stable LOX-1 NTFs are generated. [3] The LOX-1 ICD that in the final step of intramembrane proteolysis of LOX-1 is produced by SPPL2a/b and liberated into the cytosol. Especially for the LOX-1 ICD its functional role remains elusive. Typically, ICDs generated by intramembrane proteolysis are thought to migrate to the nucleus where they can affect the cellular transcriptome by either acting as a transcription factor themselves or modulating the activity of other transcription factors. While this has been unambiguously demonstrated for other RIP substrates including the γ-secretase substrate Notch-1 (93) and the site-2-protease substrate SREBP (94), in the case of intramembrane proteolysis by SPP/SPPL family members analysis of ICD function is severely hampered by the poor stability of these fragments. Mostly, SPPL substrate ICDs are rather small with a size below 100 amino acids, in the case of murine LOX-1 ~31 amino acids, which complicates their biochemical detection. Also, intramembrane proteolysis of SPP/SPPL substrates is often coupled to the subsequent proteasomal degradation of the released ICDs (69, 95). Therefore, further studies will be needed to show whether the small LOX-1 ICD exerts a transcriptional function or if it is too instable to be functionally relevant.

### The LOX-1 NTF Controls LOX-1 Signaling

LOX-1 NTFs can be detected even under basal conditions at a relevant level and therefore might participate in the regulation of LOX-1 receptor function. Indeed, upon overexpression, the LOX-1 NTF is able to form oligomers with other NTF molecules but also the full length receptor (69). Along this line, inhibition of SPPL-dependent processing of LOX-1 leads to an increased ERK1/2 activation upon stimulation of LOX-1 expressing cells with oxidized LDL particles indicating that NTF levels might indeed influence the pro-atherogenic signaling cascade of LOX-1 by directly binding to the full length receptor. This putative pro-atherogenic function of the LOX-1 NTF was further validated in a mouse model of atherosclerosis, where SPPL2a/b double-deficient mice subjected to a bone marrow transplantation with wild type bone marrow were treated with a high fat diet after lowering of LDLR levels by overexpression of a PCSK9 gain-of-function mutant. In this model, despite lower total cholesterol levels, SPPL2a/b double-deficient mice presented larger and more fibrotic plaques than wild type controls. In agreement with findings from cellular model systems, also here increased MAPK activation was demonstrated (69).

In addition to this, the LOX-1 NTFs – at least when present in higher amounts – also appear to be able to induce signaling independent of the full length receptor. Overexpression of a LOX-1 fragment consisting of the first 88 amino acids of the receptor induced activation of ERK1/2 and p38 MAPK in HEK cells, which did not show any detectable endogenous expression of LOX-1 (69). Even though to different levels, upregulated ERK1/2 and p38 phosphorylation was sensitive to the MEK inhibitor U0126 and the Src inhibitor Saracatinib arguing for a contribution of these upstream kinases. In contrast, no activation of the NFκB and Akt pathways was observed upon LOX-1 NTF overexpression. While the precise mechanism how the LOX-1 NTFs might facilitate this MAPK activation remains to be investigated, oligomerization of NTF molecules appears to be a crucial factor. Elimination of putative phosphorylation sites in the LOX-1 cytosolic domain did not influence MAPK phosphorylation. However, mutated versions of the LOX-1 NTF that showed a significantly reduced ability to form dimers with the wild type LOX-1 NTF also failed to induce ERK1/2 phosphorylation (69). Oligomerization of LOX-1 or its NTF therefore might induce a signaling competent complex that even in absence of a respective LOX-1 ligands is able to induce pro-atherogenic signaling cascades. Therefore, abundance control of LOX-1 NTFs by SPPL2a and SPPL2b can be regarded as a novel athero-protective mechanism.

### sLOX-1 as Potential Biomarker

Even though ectodomain shedding of LOX-1 was described 20 years ago and sLOX-1 can be readily detected in cell culture supernatants as well as in the blood of patients suffering from acute coronary syndrome (76, 96), the physiological function of this LOX-1 fragment remains completely elusive. In principle, sLOX-1 could act as a decoy receptor masking excessive oxLDL in the circulation, thereby preventing an overshooting atherosclerotic response of cells expressing LOX-1 or other oxLDL-binding scavenger receptors. The presence of soluble decoy receptors generated by ectodomain shedding has been demonstrated for various receptor families including Toll-like receptors (97), interleukin receptors (98) and receptor tyrosine kinases (99–101). However, so far there are no reports about the function of a shed decoy receptor from the C-type lectin receptor family. Even though there are no data published in the case of LOX-1, in their initial description of sLOX-1 Murase et al. state that in preliminary experiments sLOX-1 failed to inhibit oxLDL binding to LOX-1 expressing cells arguing against a role of sLOX-1 as decoy receptor (76). However, detailed studies analyzing a potential competition of sLOX-1 and the full length receptor for their ligand oxLDL are pending which may help to characterize the pathophysiological role of sLOX-1.

Despite the still unclear function of sLOX-1 in the circulation there is growing evidence that this receptor fragment might be used as clinical biomarker for the diagnosis of vascular disease including coronary artery disease (CAD), acute coronary syndrome (ACS), ischemic stroke and acute aortic dissection (AAD). The disease associations of sLOX-1 and its potential clinical uses were recently reviewed in a very comprehensive way (81), why we discuss these aspects here only briefly. As analyzed by classical sandwich ELISAs, in healthy individuals sLOX-1 is present in very low concentrations (few pg/ml). In contrast, patients suffering from any of the diseases described above present a robust increase in sLOX-1 levels in the range of several ng/ml (79–81, 102). sLOX-1 levels correlate with increased oxidative stress as well as vascular inflammation, plaque vulnerability and plaque fibrosis and therefore might represent an early marker of progressing atherosclerotic lesions (103).

Our understanding of the mechanisms that cause sLOX-1 liberation *in vivo* is still limited. For example, the source of sLOX-1 in the circulation is still not fully understood. As indicated before, LOX-1 is expressed by a variety of cell types crucial for the development and progression of atherosclerotic plaques. In addition, LOX-1 is also present in several other cell types including cardiomyocytes where it is also upregulated by disease-associated conditions as discussed above. In acute coronary syndrome sLOX-1 appears to be a rather early marker and the increase of sLOX-1 preceded the release of classical markers of cardiomyocyte damage like cardiac troponin T (96). In atherosclerotic plaques, LOX-1 expression correlates with presence of activated endothelial cells as well as macrophages (104). Nevertheless, the contribution of these cells to the total release of sLOX-1 has not been determined. The upregulation of LOX-1 under several pathophysiological conditions is well-documented. However, sLOX-1 levels do not directly correlate with plaque LOX-1 mRNA in carotid atherosclerosis (104). While this might indicate sLOX-1 release from non-plaque tissue, it also highlights the necessity to identify the physiologically relevant proteases responsible for this process. Ectodomain shedding typically is a tightly regulated event in which elevated substrate transcription does not automatically correlate with increased shedding activity. Several metalloproteinases belonging to the MMP and ADAM family have been demonstrated to be more active under inflammatory conditions (105, 106) and therefore also might be responsible for elevated liberation of sLOX-1 from stimulated vascular cells. However, so far the contribution of elevated *Olr1* transcription and LOX-1 shedding to the increase of sLOX-1 in patients with cardiovascular disease remains elusive. In summary, a more detailed analysis of the molecular pathways crucial for the increase of sLOX-1 in cardiovascular disease in combination with large-scale clinical studies is required to further establish this receptor fragment as early biomarker for CAD, ACS, AAD, and stroke.

## Conclusions And Open Questions

Genetic ablation of LOX-1 in mice has major beneficial effects in different models of cardiovascular diseases. Based on this critical pathophysiological role, LOX-1 has to be considered as a therapeutic target. Inhibition of LOX-1 functions with regard to oxLDL uptake and pro-atherogenic signaling would be expected to slow down disease progression. An obvious approach in order to target the LOX-1 receptor would be to inhibit binding of ligands and thereby also block downstream responses. Indeed, small molecule inhibitors of LOX-1 identified either by high throughput screening (107) or structure-based design (108) have been developed. However, so far these compounds were only characterized in cellular systems and evaluation *in vivo* has not been reported yet.

Perspectively, downstream and/or regulatory pathways may also be considered as alternative points to target LOX-1. Currently, proximal events triggered by LOX-1 activation which lead to the activation of effector pathways like MAP kinases are poorly characterized making it impossible to judge the potential of pharmacologic interventions at these steps. Amongst other mechanisms, proteolysis is emerging as a central regulatory mechanism of LOX-1 function acting at different levels. Based on our current understanding, it may be assumed that increased LOX-1 proteolysis has an attenuating effect on atherosclerotic plaque development. Increased ectodomain shedding reduces the surface receptor which should limit the oxLDL uptake capacity and also the pro-atherogenic signaling of the receptor. Similarly, in light of the autonomous signaling function of LOX-1 NTF, an increased SPPL2a/b activity may be considered to be anti-atherogenic. However, these simplistic considerations are associated with several open questions. Since no biological function of sLOX-1 is known, it cannot not fully be excluded that an overproduction of this fragment has adverse effects. In any case, identification of the responsible sheddase *in vivo* would represent an essential prerequisite to assess potential strategies to activate this process. Though with regard to the intramembrane cleavage of the LOX-1 NTF the proteases have been identified, very little is known about the regulation of SPP/SPPL proteases. Therefore, it is currently elusive how an increased cellular activity of these proteases could be achieved. In general, in all these considerations it has to be kept in mind that proteases have several substrates and that it is usually not possible to increase proteolytic activity just toward one particular substrate. At the moment, it cannot be excluded that a globally increased activity of SPPL2a or SPPL2b or the sheddase of LOX-1 is associated with adverse effects due to the upregulated cleavage of other substrates. Further studies on LOX-1 itself but also on the involved proteases will be needed to evaluate these open questions and to assess the translational potential of the pathways described. In light of its general potential as therapeutic target, a deeper understanding how proteolytic regulation LOX-1 impacts on disease processes will be of critical importance.

## Author Contributions

All authors listed have made a substantial, direct and intellectual contribution to the work, and approved it for publication.

## Conflict of Interest

The authors declare that the research was conducted in the absence of any commercial or financial relationships that could be construed as a potential conflict of interest.
